# Supplementary material for: Doubly Dynamic Hydrogel Formed by Combining Boronate Ester and Acylhydrazone Bonds
Source: Polymers (Basel). 2020 Feb 21;12(2):487. doi: 10.3390/polym12020487 (PMC7077654; doi:10.3390/polym12020487)
Supplement: Supplementary file 1 [file polymers-12-00487-s001.pdf]

Supporting Information for

Doubly Dynamic Hydrogel Formed by Combining Boronate Ester and Acylhydrazone Bonds

Yusheng Liu [1], Yigang Liu [2], Qiuxia Wang [2], Yugui Han [2], Hao Chen [1,*] and Yebang Tan [1,*]

[1] School of Chemistry and Chemical Engineering, Key Laboratory of Special Functional Aggregated Materials, Ministry Education, Shandong University, Jinan 250100, People's Republic of China; liuyusheng@mail.sdu.edu.cn (Y.L)

[2] CNOOC, Ltd., Tianjin Branch, Bohai Oilfield Research Institute, Tanggu Tianjin 300452, People's Republic of China; liuyg@cnooc.com.cn (Y.L); wangqx17@cnooc.com.cn (Q.W); hanyg4@cnooc.com.cn (Y.H)

* Corresponding author. chenh@sdu.edu.cn (H.C); ybtan@sdu.edu.cn (Y.T); Tel.: +86-531-88363502 (H.C); +86-531-88363502 (Y.T); Fax: +86-531-88564464 (H.C); +86-531-88564464(Y.T)

1. Experimental Section

1.1. Synthesis of N-acryloyl-3-aminophenylboronic acid (AAPBA)[1]

3-aminophenylboronic acid (5.478 g) was dissolved in 80 mL of 2 M NaOH and cooled in a low-temperature reactor at –5 °C with magnetic stirring for 10 min. Subsequently, acryloyl chloride (5.20 mL) was added dropwise. The reaction mixture was reacted at –5 °C for 30 min and then was carried out at room temperature for another 120 min, followed by the reaction mixture being adjusted the pH to 2 with 2 M HCl solution. The precipitate was filtered off and washed three times with 25 mL of cold deionized water. The precipitate was dissolved in hot water (70 °C) and filtered off the insoluble impurities. The hot filtrate was left to a refrigerator overnight and the product was obtained by filtered off. The yield of AAPBA was 69.8%.

^{1}H NMR (300 MHz, DMSO-d_6) δ 10.08 (d, *J* = 16.0 Hz, 1H), 8.01 (s, 2H), 7.94 – 7.77 (m, 2H), 7.50 (dt, *J* = 7.3, 1.2 Hz, 1H), 7.30 (q, *J* = 8.5, 7.7 Hz, 1H), 6.45 (dd, *J* = 17.0, 10.0 Hz, 1H), 6.24 (dd, *J* = 17.0, 2.2 Hz, 1H), 5.73 (dd, *J* = 10.0, 2.2 Hz, 1H).

1.1. Synthesis of P(NIPAM-co-DPA)

The P(NIPAM-*co*-DPA) (PD) was prepared from NIPAM and DAA through RAFT polymerization using DDMAT as a chain transfer agent (CTA). Take the preparation of copolymer PB1 in Table 1 as an example. NIPAM (5.09 g, 45 mmol), DAA (0.85 g, 3.0 mmol), DDMAT (0.365 g, 0.1 mmol), AIBN (3.3 mg, 0.02 mmol) were dissolved in 15.5 mL DMSO in a 25 mL reaction tube flask equipped with a stir bar. The mixture was degassed via three freeze-pump–thaw cycles, and then the tube was immersed in a 70 °C oil bath. The copolymerization was performed for 24 h and terminated by rapid cooling upon immersion of the flask in liquid nitrogen. The solution was transferred to a semi permeable membrane (MWCO 1000) and dialyzed extensively against methanol for four days with repeated changing of methanol. The product was obtained by rotary evaporation and dried under vacuum. The other copolymers (PD1, PD3) were prepared by following the same procedure, but with different molar feed ratios of monomers.

2. Additional Data

2.1. GPC Tests

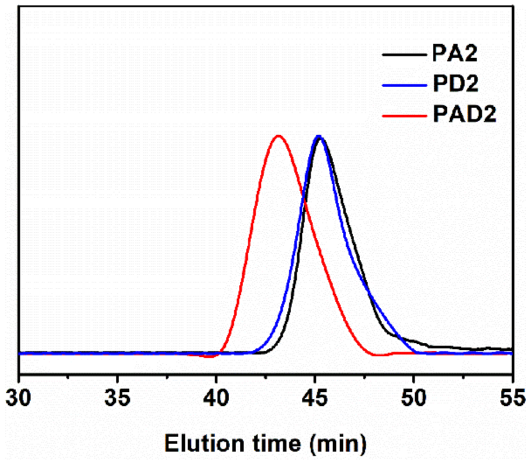

Figure S1. GPC spectra of copolymers.

2.1 *Characterization of Copolymers*

Table 1. Characterization of copolymers.

	[M]/[CTA]/[I]	Feed Ratio (%)	Conversion[a] (%)	Calculated Ratio[b] (%)	$M_{n\ theo}$[c]	M_n[d]	M_w[d]	PDI[d]
PA1	50: 1: 0.2	47: 3[e]	87.1	41: 2.3[e]	5444	4956	5642	1.14
PA2	50: 1: 0.2	45: 5[e]	84.7	38: 4.7[e]	5562	4683	5481	1.17
PA3	100:1:0.2	90: 10[e]	89.2	81: 8.0[e]	11058	11435	13144	1.22
PAD 1	50: 1: 0.2	47: 3[f]	89.9	37: 2.1[f]	9986	12548	16688	1.33
PAD 2	50: 1: 0.2	45: 5[f]	94.0	39: 4.1[f]	10688	13563	17360	1.28
PAD 3	100: 1: 0.2	90: 10[f]	91.6	73: 7.0[f]	21568	26500	35856	1.35
PD1	50: 1: 0.2	47: 3[g]	90.5	40: 2.6[g]	5331	5852	6633	1.13
PD2	50: 1: 0.2	45: 5[g]	87.4	38: 4.2[g]	5375	5158	5874	1.14
PD3	100: 1: 0.2	89: 10[g]	87.0	76: 7.4[g]	10217	10311	12579	1.19

[a] Calculated from gravimetric analysis.

[b] Calculated from ^1H NMR spectroscopy and monomer conversion.

[c] Calculated from ^1H NMR spectroscopy and monomer conversion.

[d] Obtained by GPC measurements.

[e] Molar ratios of NIPAM to AAPBA in PA.

[f] Molar ratios of NIPAM to DAA in second segment of PAD.

[g] Molar ratios of NIPAM to DAA in PD.

2.2. *Thermo-Responsive Properties*

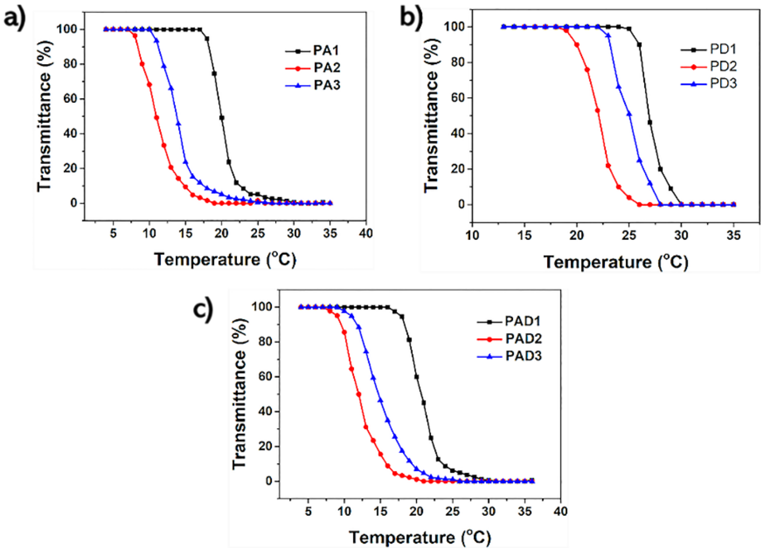

Figure S2. Thermo-responsiveness of (**a**) PA; (**b**) PD; and (**c**) PAD. The polymer concentration is 0.5 mg mL^{-1}.

PNIPAM is known for its thermo-responsive properties, such as low critical solubility temperature (LCST) in water (~32 °C) [2]. The LCSTs of PA1, PA2, and PA3 are 20.4, 11.8, and 14.1 °C, respectively. Meanwhile, the LCSTs of PD1, PD2, and PD3 are 28.2, 25.7, and 22.8 °C, respectively. The results indicated that the PAAPBA is more hydrophobic than PDAA in copolymer. The LCSTs of the diblock copolymers PAD1, PAD2, and PAD3 are 20.4, 12.1, and 14.3 °C at pH 7.0, respectively, which are closed to the LCSTs of PA1, PA2, and PA3.

2.3. pH-Responsive Properties

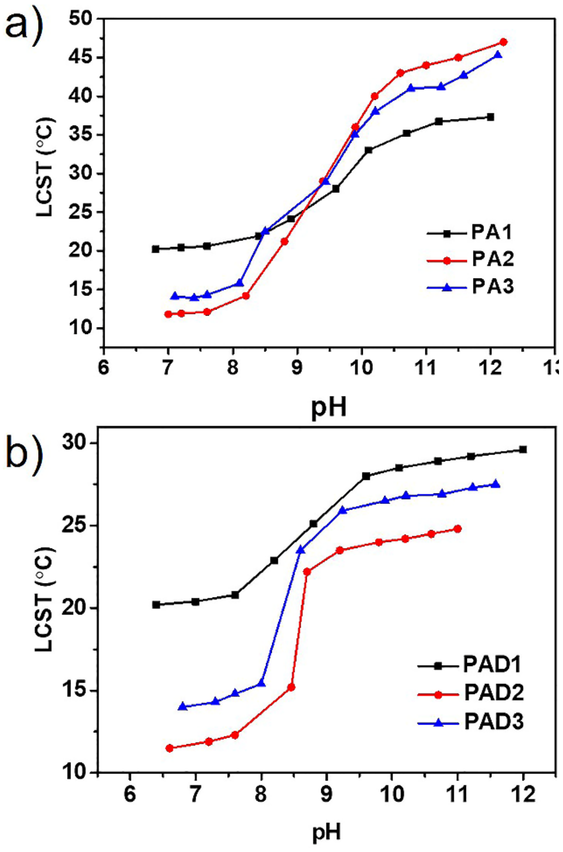

Figure S3. LCST of copolymers as a function of the pH; (**a**) PA and (**b**) diblock PAD copolymers.

The increase of pH led to the increase of LCSTs due to the transformation of the PBA group from hydrophobic to hydrophilic. The LCST of the PA copolymer reached a platform near the pH 11. In this case, most of the PBA groups have transformed. However, with the pH exceeding 9.0, the LCST curve of the diblock copolymer PAD caused a platform to appear, which may have resulted from effect of the second segment PD.

2.4. Fructose-Responsive Properties

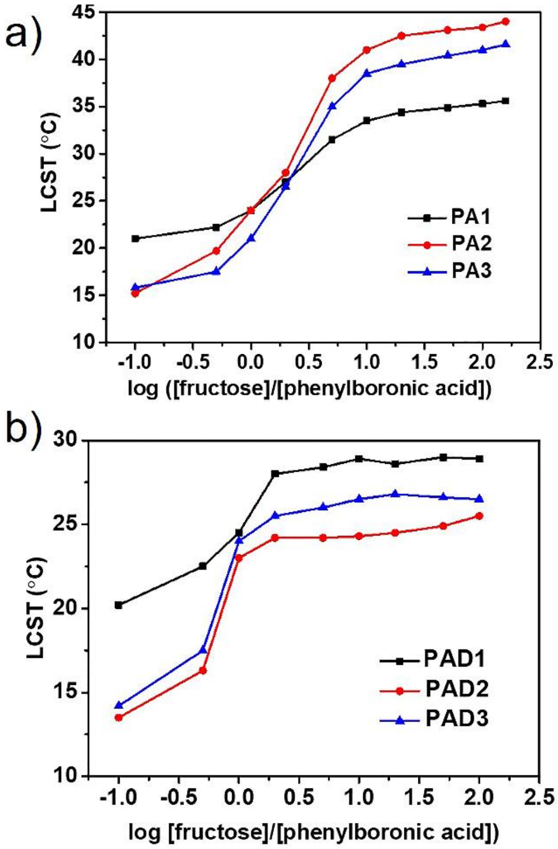

Figure S4. LCST of copolymers as a function of the log ([fructose]/[boronic acid]); (**a**) PA and (**b**) diblock PAD copolymers.

The addition of glucose also led to an increase in the LCSTs due to the transformation of the PBA group from hydrophobic phenylboronic acid to hydrophilic phenylboronate ester. However, with the log ([fructose]/[PBA]) exceeding 0.25, the LCST curve of the diblock copolymer PAD caused a platform to appear, which also could have resulted from effect of the second segment PD. The results indicated that the diblock PAD copolymers have temperature-, fructose-, and pH-responsive properties. Meanwhile, their LCSTs can be controlled by varying the composition of the copolymers, the environmental pH, and the fructose concentrations.

2.5. Gelation Kinetics of Acylhydrazone-Crosslinked Hydrogels

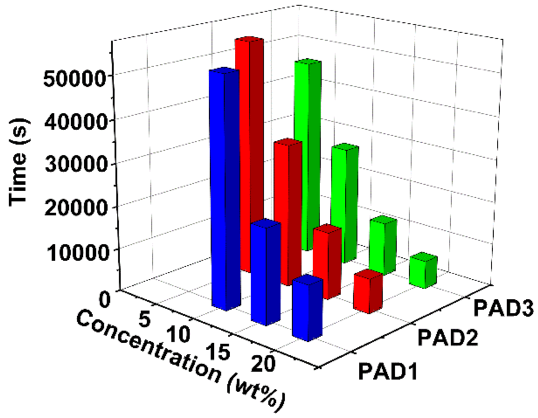

Figure S5. Crossover time of storage and loss modulus of the time sweep experiment for acylhydrazone-crosslinked hydrogels with different diblock copolymers and polymer concentrations with the molar ratios of ketone to hydrazine fixed to 1.

2.6. Oscillatory Time Sweep

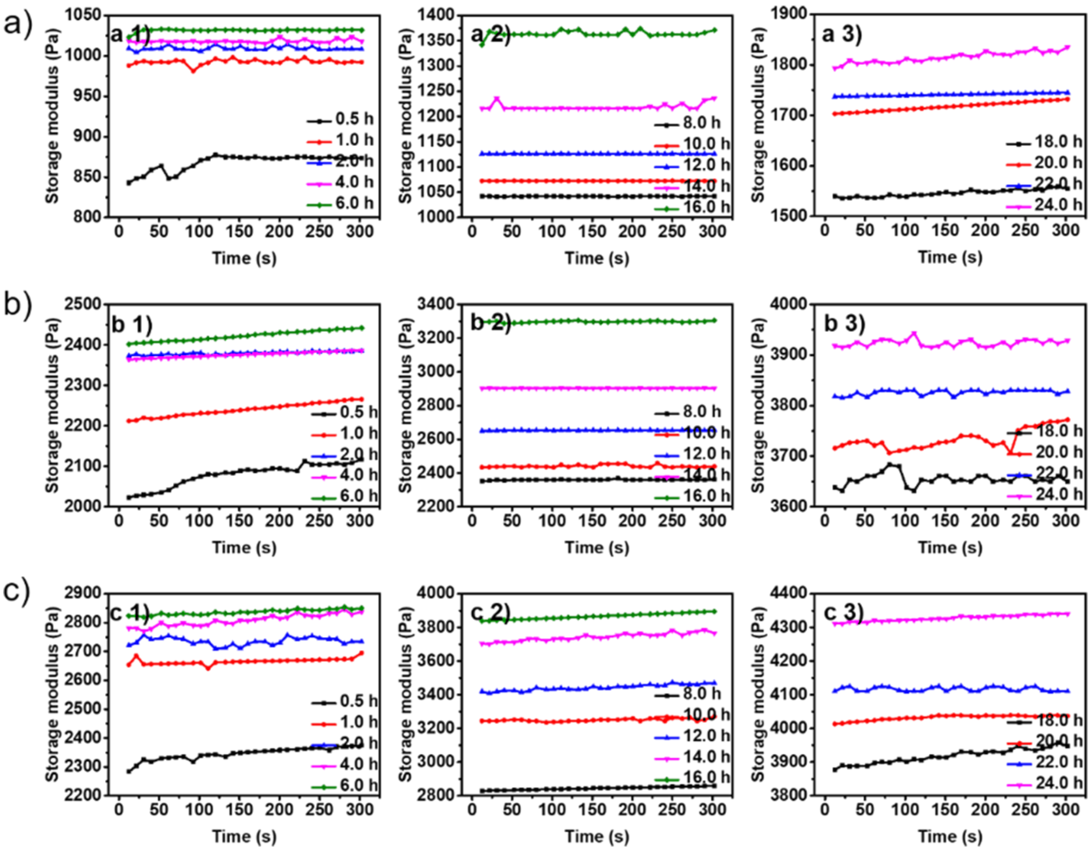

Figure S6. Oscillatory time sweep experiments of the double DCBs-crosslinked hydrogels at different time during gelation (**a**) PAD1@PVA@ADH; (**b**) PAD2@PVA@ADH; and (**c**) PAD3@PVA@ADH, the PAD2 concentration are 10 wt%, the molar ratios of the ketone to hydrazine are fixed to 1.0, and the mass ratios of the PVA to PAD2 are fixed to 0.5. All of the experiments were performed at a frequency of 1.0 Hz and a strain of 1.0%.

2.7. Storage Modulus during the Gelation

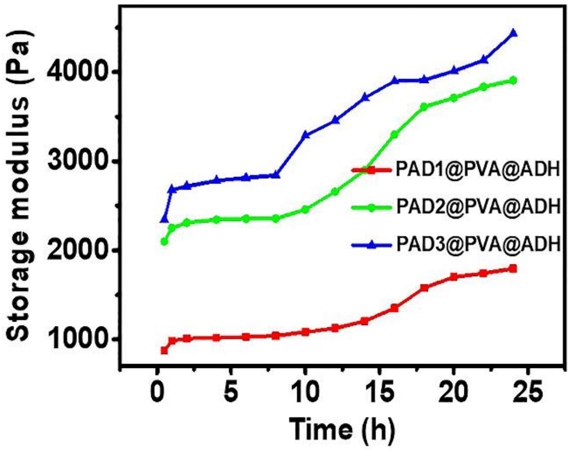

Figure S7. Storage modulus of the double DCBs-crosslinked hydrogels at different time during the gelation. The PAD2 contents of the hydrogels are fixed to 10 wt%, the molar ratios of the ketone to hydrazine were 1.0, and the mass ratios of the PVA to PAD2 are fixed to 0.5. All of the experiments were performed at a frequency of 1.0 Hz and a strain of 1.0%.

2.8. Dynamic Oscillatory Angular Frequency Sweep

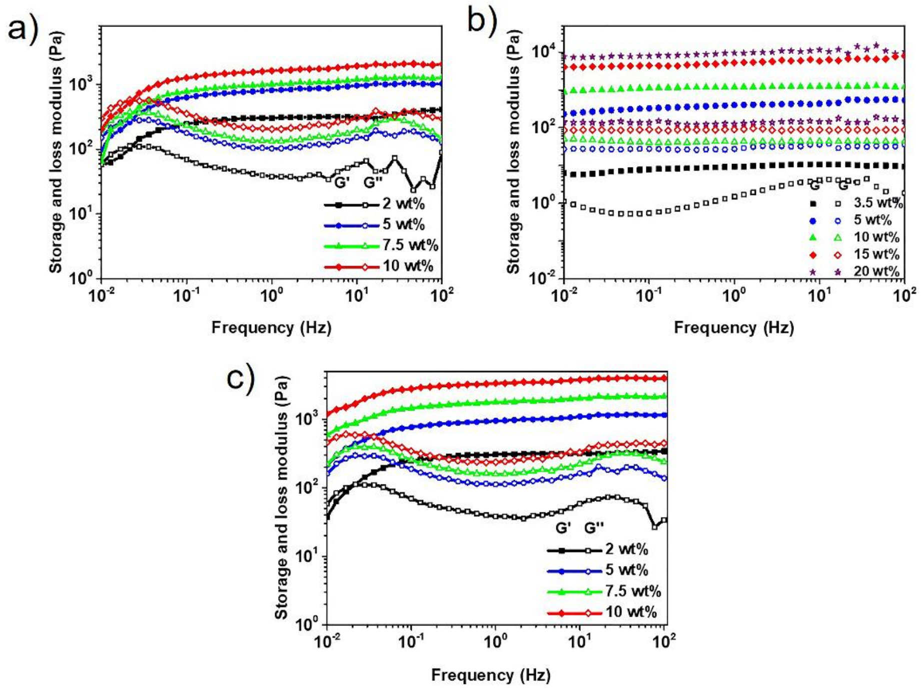

Figure S8. Dynamic oscillatory angular frequency sweep of hydrogels with various copolymer concentration; (**a**) single boronate ester-crosslinked hydrogels with the mass ratios of the PVA to PAD2 are fixed to 0.5; (**b**) single acylhydrazone-crosslinked hydrogels with the molar ratios of the ketone to hydrazine are fixed to 1.0; (**c**) double DCBs-crosslinked hydrogels with the molar ratios of the ketone to hydrazine are fixed to 1.0, and the mass ratios of the PAD to PVA are fixed to 0.5.

2.9. Mechanical Property

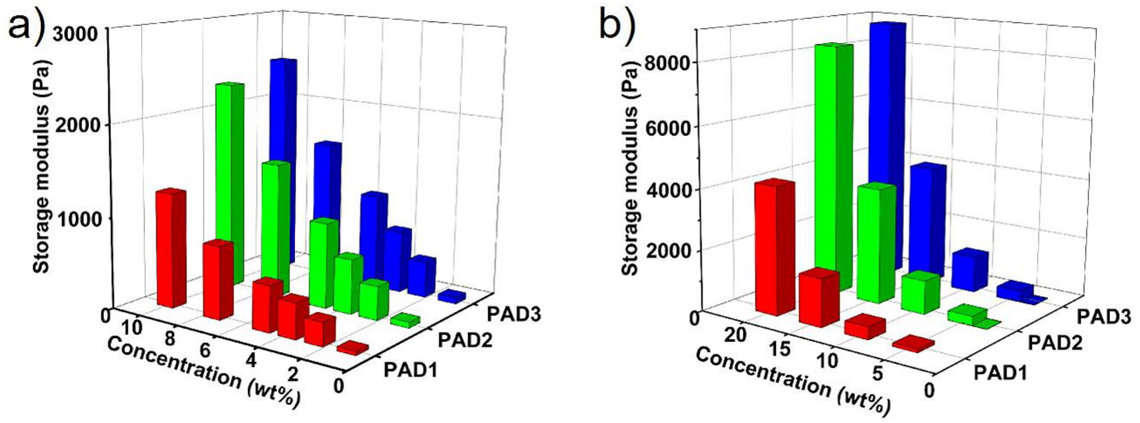

Figure S9. Storage modulus of the hydrogels with different compositions and polymer concentrations; (**a**) single boronate ester-crosslinked hydrogels the mass ratios of the PAD to PVA are fixed to 0.5; (**b**) single acylhydrazone-crosslinked hydrogels with the molar ratios of the ketone to hydrazine are fixed to 1.0. All of the experiments were performed at a frequency of 1.0 Hz and a strain of 1.0%.

2.10. Self-Healing Property

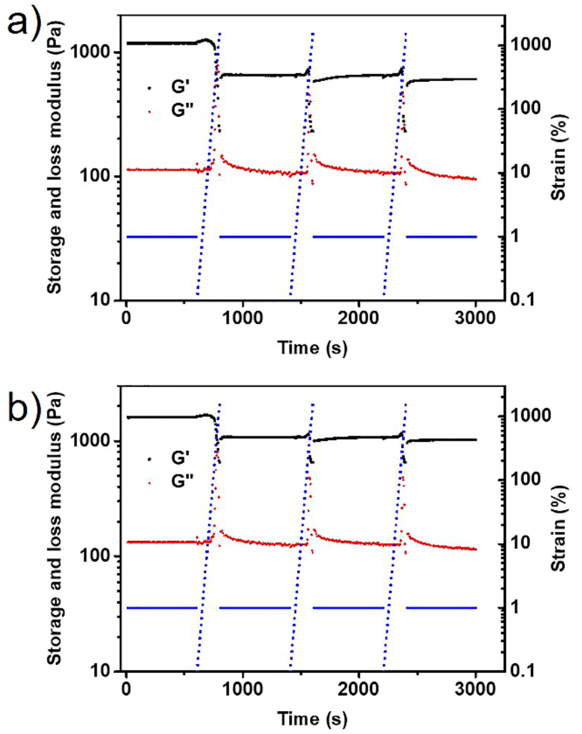

Figure S10. Repeated dynamic strain sweep tests of PAD2@PVA@ADH hydrogels with a frequency of 1.0 Hz, the PAD2 concentration is fixed to 10 wt% and the molar ratio of ketone to hydrazine is fixed to 1.0, the mass ratios of PVA to PAD2 is 0.1 for (**a**) and 0.25 for (**b**).

2.11. Self-Healing Ability

Table S2. Healing efficiency (*HE*) of the hydrogel after each cycle.

	Cycle 1 (%)	Cycle 2 (%)	Cycle 3 (%)
PAD2@ADH	45.0	42.2	39.6
PAD2@PVA	100.7	103.5	104.6

PAD2@PVA@ADH (0.1[a])	55.2	54.4	51.5
PAD2@PVA@ADH (0.25[a])	67.4	66.4	64.1
PAD2@PVA@ADH (0.5[a])	91.6	88.4	85.6

[a] Mass ratio of PVA to PAD2.

2.12. pH-Triggered Gel-Sol-Gel Phase Transitions

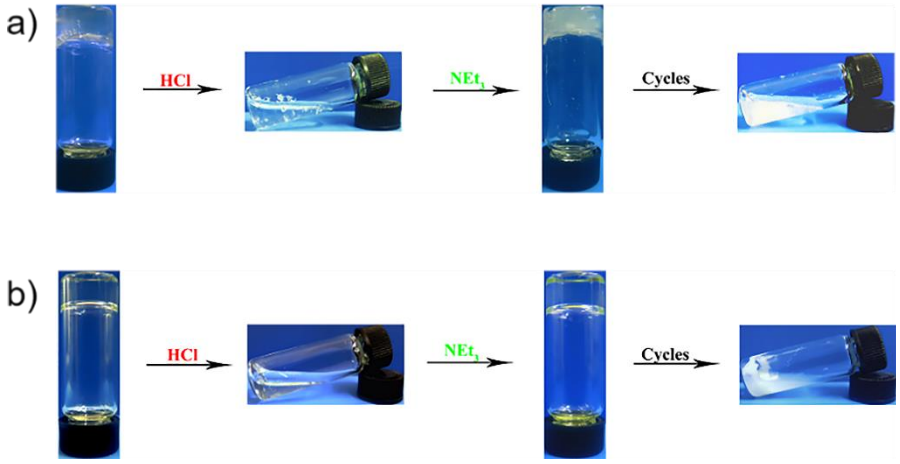

Figure S11. Photographs of the process of pH-triggered gel-sol-gel phase transitions of (**a**) PAD2@PVA; (**b**) PAD2@ADH.

2.13. Stimulus Responsiveness

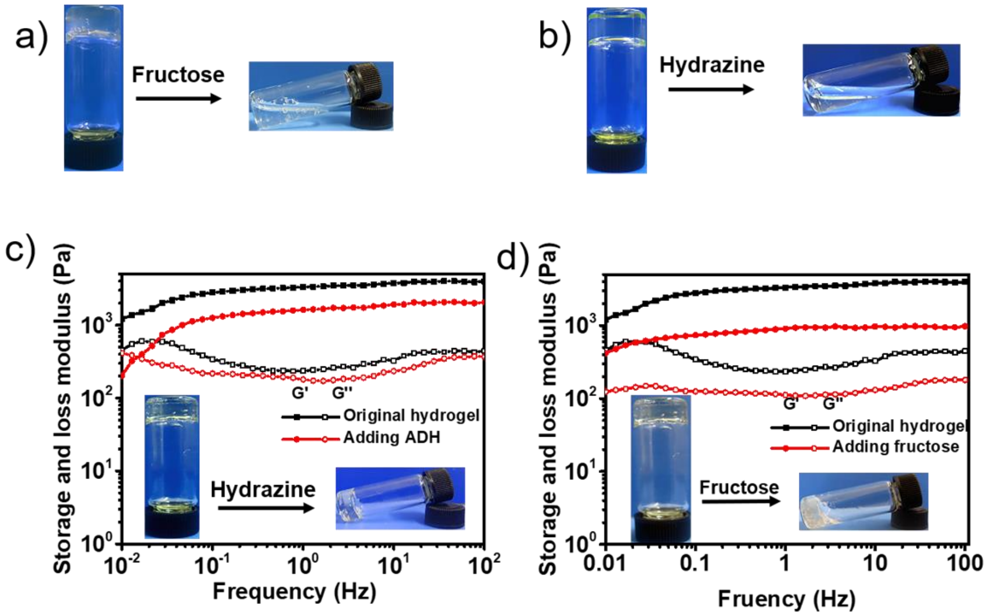

Figure S12. (**a**) fructose responsive behaviors of the boronate ester-crosslinked PAD2@PVA hydrogel; (**b**) hydrazine responsive behaviors of acylhydrazone-crosslinked PAD2@ADH hydrogels. Variation of the chemical property of the PAD2@PVA@ADH gel after being treated by (**c**) ADH and (**d**) fructose.

2.14. SEM Images

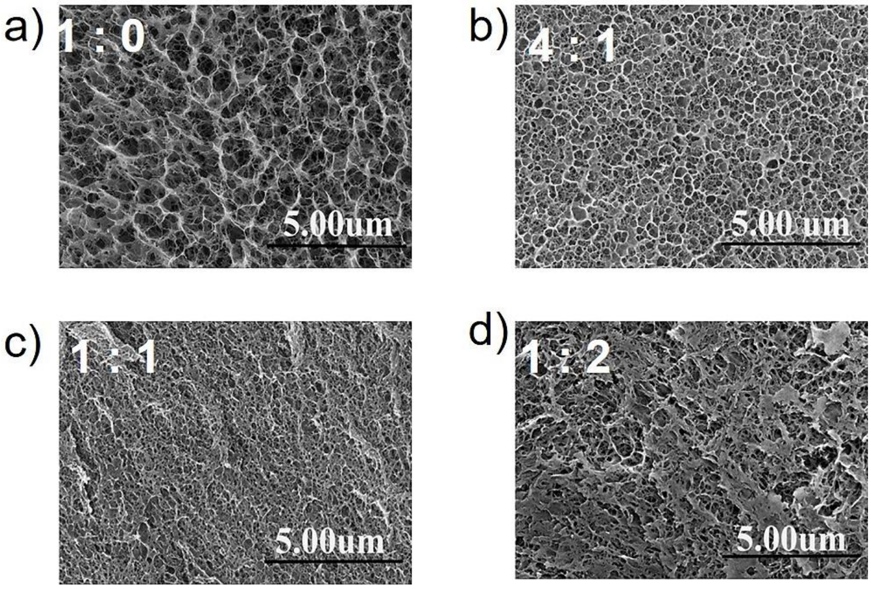

Figure S13. SEM images of the dry PAD2@PVA@ADH hydrogels with different [ketone]/[hydrazine] molar ratios (the PAD2 contents are fixed to 10 wt% and the the mass ratios of PVA to PAD2 are fixed to 0.1 for all of the samples).

1. References

1. Lee, M-C.; Kabilan, S.; Hussain, A.; Yang, X.; Blyth, J.; Lowe, C.R. Glucose-Sensitive Holographic Sensors for Monitoring Bacterial Growth. *Analytical Chemistry* **2004**, *76*, 5748-5755, doi:10.1021/ac049334n.
2. Kotsuchibashi, Y.; Ebara, M.; Yamamoto, K.; Aoyagi, T. "On–off" switching of dynamically controllable self-assembly formation of double-responsive block copolymers with tunable LCSTs. *Journal of Polymer Science Part A: Polymer Chemistry* **2010**, *48*, 4393-4399, doi:doi:10.1002/pola.24226.
